# Severe Pulmonary-Renal Syndrome Due to Hydralazine-Induced ANCA-Associated Vasculitis

**DOI:** 10.7759/cureus.111955

**Published:** 2026-07-02

**Authors:** Ayomide O Gbenle, Puneet Bedi, Lena Ibrahim, Samuel Spitalewitz, Bakhtyar Khan

**Affiliations:** 1 Nephrology, Brookdale University Hospital Medical Center, Brooklyn, USA; 2 Internal Medicine, Brookdale University Hospital Medical Center, Brooklyn, USA

**Keywords:** anca-associated vasculitis, cyclophosphamide therapy, dialysis dependent, high dose corticosteroids, hydralazine induced anca vascultis, necrotizing and crescentic glomerulonephritis, pauci-immune crescentic glomerulonephritis, plasmapheresis, pulmonary-renal syndrome, rituximab therapy

## Abstract

Antineutrophilic cytoplasmic antibodies glomerulonephritis (ANCA-GN) is the most common type of crescentic glomerulonephritis in the elderly. It is life-threatening, and early detection is key to facilitating complete remission with treatment. Albeit rare, more reports are implicating hydralazine use in the development of ANCA-associated vasculitis (AAV) and ANCA-GN. Classic treatment involves the use of corticosteroids with cyclophosphamide (+/- plasmapheresis) in ANCA-GN. However, a definite therapeutic approach and duration for drug-induced AAV has yet to be studied in clinical trials. In our case, we aim to provide another approach that would one day improve and structure therapy guidelines for drug-induced AAV.

We present a 75-year-old female patient, with medical history of hypertension, atrial fibrillation status post ablation on anticoagulation, pulmonary hypertension, who came to the nephrology clinic with symptoms of fatigue, anorexia, nausea, weight loss, hemoptysis, and rise in serum creatinine to 2.39 mg/dL from baseline of 0.65 mg/dL, and by admission to the hospital in August 2024 was 4.6 mg/dL. Anti-MPO and anti-histone antibodies were positive; complement was normal. Renal biopsy confirmed pauci-immune necrotizing GN with crescents. Therapy included steroids, rituximab, cyclophosphamide, and plasmapheresis with clinical improvement and complete remission of extra-renal disease.

## Introduction

ANCA-associated vasculitis (AAV) is the most common cause of rapidly progressive glomerulonephritis (RPGN) in older adults and carries substantial morbidity and mortality when complicated by pulmonary-renal syndrome (PRS). PRS is characterized by crescentic glomerulonephritis and diffuse alveolar hemorrhage, often requiring intensive care and renal replacement therapy.

While most cases of AAV are idiopathic, drug-induced forms have been increasingly recognized [1]. Hydralazine, a commonly prescribed antihypertensive medication, is a rare but steadily increasing documented trigger of ANCA-associated vasculitis [1]. Hydralazine-induced AAV often presents high titers of anti-myeloperoxidase (MPO) antibodies and anti-histone antibodies and may demonstrate overlapping features of drug-induced lupus [2,3]. Approximately two-thirds of hydralazine-induced AAV patients have been found to have pulmonary involvement in addition to renal dysfunction [2]. Optimal induction therapy for severe hydralazine-induced PRS remains unclear [4], particularly in patients with advanced renal failure or respiratory failure requiring mechanical ventilation.

We report a case of severe pulmonary-renal syndrome secondary to hydralazine-induced AAV treated with corticosteroids, rituximab, cyclophosphamide, and plasmapheresis, contributing to the limited data guiding management in this high-risk population.

## Case presentation

A 75-year-old woman with a history of hypertension, atrial fibrillation status post ablation on chronic anticoagulation, and pulmonary hypertension was referred to the nephrology clinic for evaluation of rapidly worsening kidney function. Her baseline serum creatinine was 0.65 mg/dL (normal 0.6-1.2 mg/dL) in May 2023. In August 2024, her creatinine rose to 2.8 mg/dL (normal 0.6-1.2 mg/dL) and progressed to 4.6 mg/dL (normal 0.6-1.2 mg/dL) by the time of hospital admission at the end of August.

The patient reported several months of progressive fatigue, anorexia, nausea, and unintentional weight loss. Her history did not highlight any inciting event for the development of such an accelerated decline in renal function, necessitating the inclusion of rapidly progressive glomerulonephritis in the differential diagnosis. Two weeks prior to admission, she developed hemoptysis, prompting further evaluation. Review of her medication history revealed long-term hydralazine use, initially started in 2015 and progressively increased to a dose of 100 mg three times daily by 2019 as part of her antihypertensive regimen.

**Figure 1:**
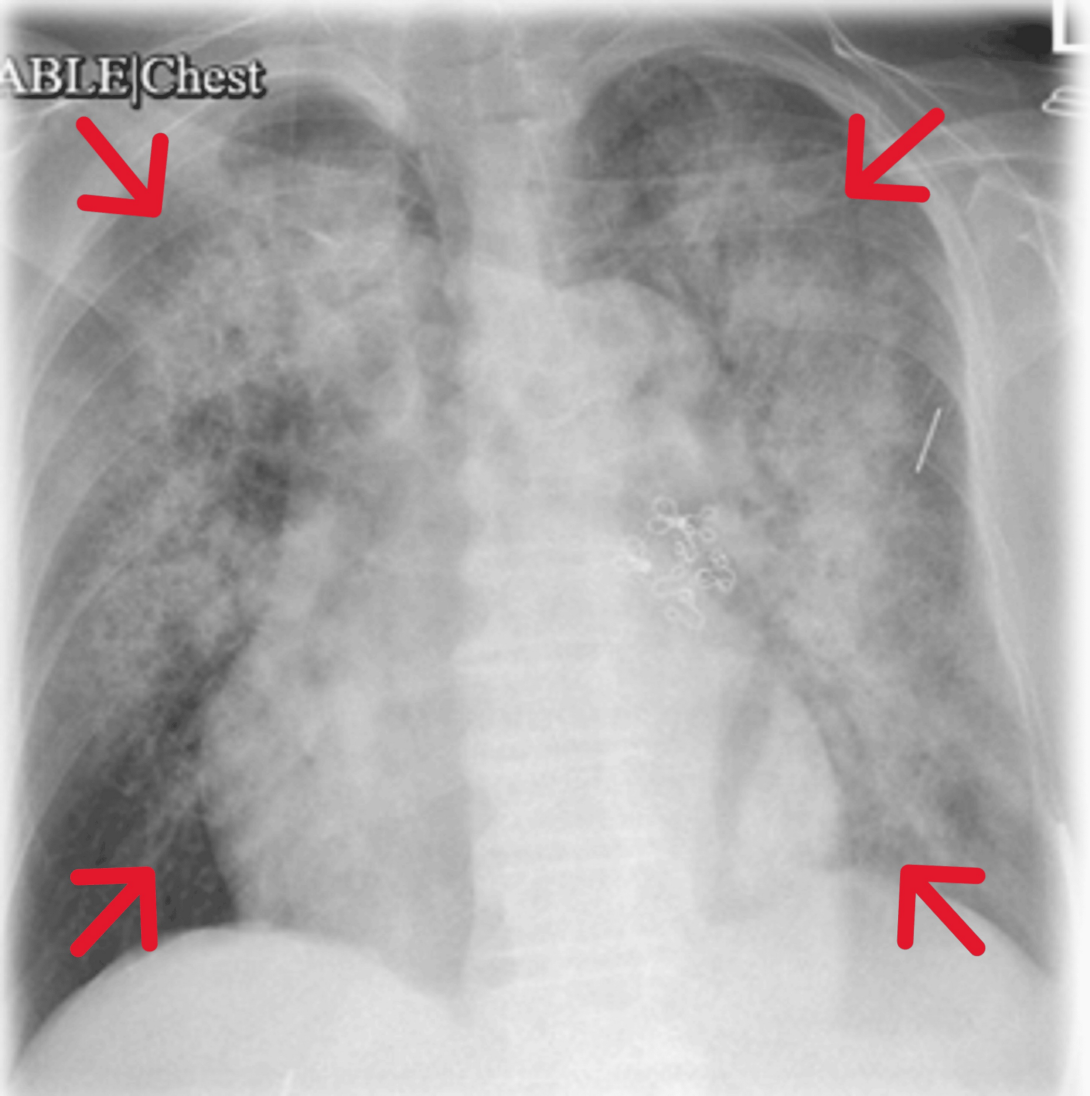
Portable AP chest radiograph Demonstrating bilateral pulmonary infiltrates (red arrows) consistent with diffuse alveolar hemorrhage.

On admission, laboratory studies demonstrated severe acute kidney injury (BUN/Cr 51/4.6 mg/dL (normal 7-25/0.6-1.2 mg/dL) with active urinary sediment (3+ protein, 3+ blood, WBC 21-50/hpf, and RBC >20/hpf on urinalysis). Her physical examination was mostly non-contributory except for a few scattered expiratory wheezes. Serologic evaluation revealed positive anti-MPO ANCA at 3.1 units (normal 0.0-0.9 units) and anti-histone antibodies at 1.1 units (normal <1.0 units), with normal complement levels. Other autoimmune and infectious etiologies, including anti-nuclear antibodies, anti-dsDNA, anti-Smith, anti-RNP, anti-SSA, anti-SSB, and anti-GBM antibodies, were negative. Chest imaging demonstrated findings consistent with pulmonary hemorrhage (Figure 1).

**Figure 2:**
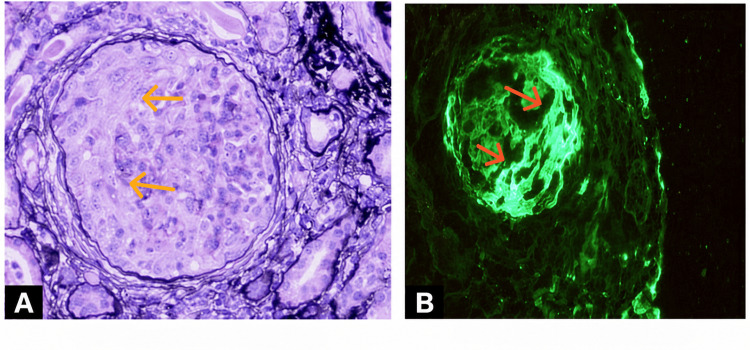
Silver methenamine staining on light microscopy (A) and immunofluorescence microscopy (B). (A) Glomerulus demonstrating crescent formation (yellow arrows) on light microscopy. (B) Fibrinogen immunofluorescence highlighting crescent formation (orange arrows).

**Figure 3:**
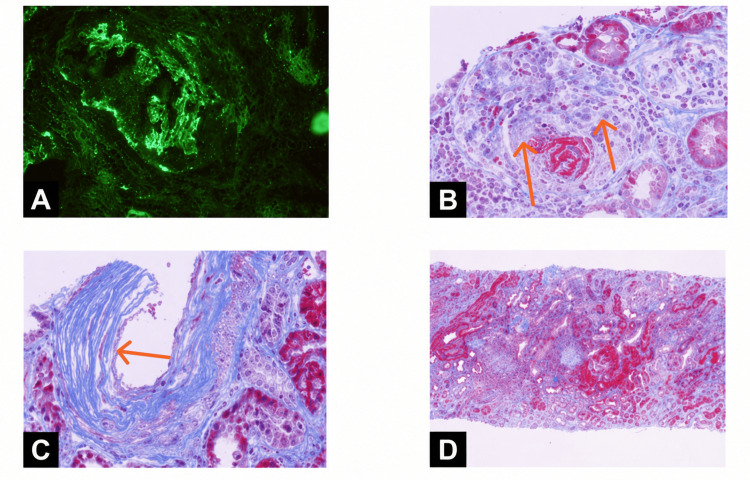
(A) Immunofluorescence microscopy (A) and trichome stain on light microscopy (B-D). (A) Rare trace capillary loop staining for C3 on immunofluorescence microscopy demonstrating pauci-immune staining (only minimal to absent glomerular immunoglobulin/complement deposition, characteristic of ANCA-associated vasculitis). (B) Segmental fibrinoid necrosis (orange arrows) identified within a glomerulus, with obliteration of the glomerular tuft by extracellular matrix, representative of chronic irreversible glomerular injury. (C) Moderate intimal sclerosis and medial thickening of arteries and arterioles (orange arrows), reflecting chronic vascular remodeling accompanying advanced renal injury. (D) Severe chronic interstitial fibrosis and tubular atrophy (IFTA), with scattered globally sclerosed glomeruli, consistent with the chronic irreversible changes.

At the time of renal biopsy, renal recovery was not expected as her serum creatinine was significantly elevated to 4.0 mg/dL (normal 0.6-1.2 mg/dL). Renal biopsy revealed findings consistent with pauci-immune necrotizing crescentic glomerulonephritis. There were crescents identified in 27 of 78 glomeruli, with segmental fibrinoid necrosis in 6 of 78 glomeruli, and global glomerulosclerosis in 48 of 78 glomeruli on light microscopy. On immunofluorescence, 19 of 35 glomeruli were globally sclerosed, without definitive glomerular staining for IgG, IgA, IgM, C1q, or albumin. Fibrinogen, however, highlighted 17 crescents (Figures 2, 3). On electron microscopy, there was near global effacement of foot processes, without significant mesangial, subendothelial, intramembranous, or subepithelial electron-dense deposits.

In the context of prolonged hydralazine use and characteristic serologic findings, hydralazine-induced AAV, presenting severe pulmonary-renal syndrome, was diagnosed. 

Management and clinical course

Hydralazine was immediately discontinued upon diagnosis. The patient was treated with high-dose intravenous corticosteroids, followed by induction therapy with rituximab and then cyclophosphamide. Cyclophosphamide was added to the induction regimen because of retroperitoneal bleeding presumed secondary to small-vessel vasculitis that occurred despite her receiving her first dose of rituximab. Given the severity of pulmonary involvement and diffuse alveolar hemorrhage requiring intubation and mechanical ventilation, plasmapheresis was initiated.

Although her hospital course was complicated by diffuse alveolar hemorrhage, acute hypoxic respiratory failure requiring mechanical ventilation, retroperitoneal bleeding, and progressive renal failure necessitating initiation of renal replacement therapy, the patient demonstrated stabilization of pulmonary status and clinical improvement, responding to combination immunosuppressive therapy. Testing of CD19+/CD20+ B-cells at the two-week mark following rituximab administration confirmed B-cell depletion at 0.0% (normal 4.6-22.1%) and 0.0% (normal 5.0-22.3%) CD19+ B-cells and CD20+ B-cells, respectively.

Renal failure persistence at one-month post-hospitalization follow-up, despite the effectiveness of rituximab, is consistent with the severity of crescentic disease at presentation and subsequent complication by acute tubular necrosis with oliguria due to hemodynamic instability from retroperitoneal bleeding during hospitalization. ANCA titers were negative at three-month follow-up, and at six-month follow-up, there was no recurrence of pulmonary disease, and maintenance treatment with rituximab was not required. At one year post-admission, she remains hemodialysis-dependent without any signs of recurrence.

She achieved complete remission of pulmonary manifestations with one dose of rituximab and one dose of cyclophosphamide in combination with plasmapheresis.

## Discussion

ANCA-associated RPGN is a life-threatening condition that requires rapid diagnosis and aggressive treatment. Pulmonary-renal syndrome represents one of the most severe manifestations of AAV and is associated with high short-term mortality, particularly in elderly patients.

Hydralazine-induced AAV is rare but increasingly reported, particularly in elderly patients receiving long-term therapy at high cumulative doses, slightly favoring females over males [1]. Distinguishing hydralazine-induced AAV from idiopathic disease is clinically important, as drug withdrawal is a critical component of management [2,5]. Serologically, hydralazine-induced AAV is commonly associated with anti-MPO ANCA, anti-histone antibodies, as in our patient, and occasionally anti-dsDNA antibodies, with complement levels often normal to low-normal [6]. Renal biopsy typically demonstrates pauci-immune necrotizing crescentic glomerulonephritis [1], as seen in this patient.

Traditional induction therapy for severe AAV includes high-dose corticosteroids and cyclophosphamide, with plasmapheresis historically employed in cases of diffuse alveolar hemorrhage [4,7]. The RAVE trial demonstrated that rituximab is non-inferior to cyclophosphamide for induction of remission in AAV; however, patients with serum creatinine greater than 4.0 mg/dL (normal 0.6-1.2 mg/dL) or those requiring mechanical ventilation were excluded [8]. The PEXIVAS trial demonstrated that plasma exchange did not significantly reduce the incidence of end-stage kidney disease or death among patients with severe AAV, leading to a reassessment of its routine use for renal indications [9]. Consequently, evidence guiding therapy in patients with severe pulmonary-renal syndrome remains limited, particularly regarding hydralazine-induced AAV [4,6].

This case is notable for the use of combined rituximab-cyclophosphamide therapy in a patient with advanced renal failure requiring dialysis and respiratory failure requiring mechanical ventilation, features that excluded patients from major randomized trials. The patient’s clinical response following the first treatment with rituximab supports the known fact that rituximab may be a viable induction agent even in severe disease, particularly when used in combination with corticosteroids. Our addition of cyclophosphamide to her regimen was initiated due to severe, life-threatening retroperitoneal bleeding. We maintained the possibility that the bleeding could have very well been a consequence of the renal biopsy; however, the cessation of bleeding following cyclophosphamide raises consideration for small-vessel vasculitis as a significant contributing factor to her retroperitoneal bleed.

Our use of plasmapheresis in this case was not targeted at renal recovery but rather at the management of her severe and life-threatening pulmonary hemorrhage. Although PEXIVAS did not demonstrate an overall survival or renal benefit from plasma exchange, relatively few patients had severe pulmonary hemorrhage requiring mechanical ventilation, as was the case with our patient. A subsequent Bayesian reanalysis suggested that a clinically meaningful benefit in patients with alveolar hemorrhage remains plausible and cannot be excluded, supporting individualized consideration of plasma exchange in selected cases of severe pulmonary involvement [10].

Emerging observational data and case series increasingly support rituximab's use in severe renal and pulmonary involvement, but prospective studies are lacking. In our case, maintenance therapy was not required at 12-month follow-up [6], perhaps with a contribution from the inherent immunosuppressed state in a dialysis-dependent patient. Our case adds to the growing literature suggesting that a one-time combination rituximab-cyclophosphamide regimen can be considered in carefully selected patients with life-threatening pulmonary renal syndrome induced by hydralazine use, after cessation of the offending agent.

## Conclusions

Hydralazine-induced ANCA-associated vasculitis is a rare but severe complication of long-term hydralazine therapy and may present as life-threatening pulmonary-renal syndrome. In the event of severe renal disease, plasmapheresis would not be targeted at renal recovery but should be done for the treatment of the pulmonary hemorrhage. Perhaps a one-time combination of rituximab and cyclophosphamide, two weeks apart, with corticosteroids might just be sufficient to achieve complete disease remission, following cessation of the offending drug, without the need for maintenance therapy in hydralazine-induced AAV manifesting as pulmonary-renal syndrome. Clinicians should maintain a high index of suspicion in patients receiving hydralazine who develop rapidly progressive renal failure and/or pulmonary hemorrhage.

This case highlights the successful use of rituximab-cyclophosphamide induction therapy in combination with corticosteroids and plasmapheresis in severe PRS as a consequence of hydralazine-induced AAV. Further studies are needed to establish optimal treatment strategies for severe hydralazine-induced AAV, and perhaps our proposed treatment regimen could be expanded to other drug-induced AAV or prospective studies.
